# Biomechanical evaluation of three surgical scenarios of posterior lumbar interbody fusion by finite element analysis

**DOI:** 10.1186/1475-925X-11-31

**Published:** 2012-06-18

**Authors:** Zhitao Xiao, Liya Wang, He Gong, Dong Zhu

**Affiliations:** 1 State Key Laboratory of Automotive Simulation and Control, Jilin University, Changchun, People’s Republic of China; 2 Department of Engineering Mechanics, Nanling Campus, Jilin University, Changchun, 130025, People’s Republic of China; 3 Department of Orthopaedic Trauma, First Hospital of Jilin University, Changchun, People’s Republic of China

**Keywords:** Spine, Cage, PEEK, Autogenous iliac bone, Ligaments

## Abstract

**Background:**

For the treatment of low back pain, the following three scenarios of posterior lumbar interbody fusion (PLIF) were usually used, i.e., PLIF procedure with autogenous iliac bone (PAIB model), PLIF with cages made of PEEK (PCP model) or titanium (Ti) (PCT model) materiel. But the benefits or adverse effects among the three surgical scenarios were still not fully understood.

**Method:**

Finite element analysis (FEA), as an efficient tool for the analysis of lumbar diseases, was used to establish a three-dimensional nonlinear L1-S1 FE model (intact model) with the ligaments of solid elements. Then it was modified to simulate the three scenarios of PLIF. 10 Nm moments with 400 N preload were applied to the upper L1 vertebral body under the loading conditions of extension, flexion, lateral bending and torsion, respectively.

**Results:**

Different mechanical parameters were calculated to evaluate the differences among the three surgical models. The lowest stresses on the bone grafts and the greatest stresses on endplate were found in the PCT model. The PCP model obtained considerable stresses on the bone grafts and less stresses on ligaments. But the changes of stresses on the adjacent discs and endplate were minimal in the PAIB model.

**Conclusions:**

The PCT model was inferior to the other two models. Both the PCP and PAIB models had their own relative merits. The findings provide theoretical basis for the choice of a suitable surgical scenario for different patients.

## Introduction

The aims of posterior lumbar interbody fusion (PLIF) procedure using cages or bone grafts are to provide stability of the motion segment and to facilitate the fusion process. After about 60 years of development and update, the surgical scenarios with cages or autogenous iliac bone (AIB) have been widely used.

The PLIF with AIB provided high fusion rate because the AIB was histo-compatible and non-immunogenic [1,2]. However, several studies reported the major complications of this surgical method with a wide range of incidence varying between 1% and 39%, such as collapse, retropulsion of the grafted bone, and pseudoarthrosis [3-6]. To resolve such problems, the PLIF with cages was designed in 1991 [7]. The advantage of this surgical scenario was that the cages separated the mechanical and biologic functions of the PLIF. Many studies reported that the PLIF with cages could provide satisfactory clinical results [8-10]. However, this surgical scenario produced new problems such as adjacent segment degeneration (ASD), fine motion and mote of cages, and implants damage [11,12].

Recently, with the development of material industry, polyetheretherketone (PEEK) aroused wide concern. Previous studies showed that PEEK was non-resorbable and elicited minimal cellular response, intracutaneous, and intramuscular toxicity [13,14]. Both the *in vitro* and finite element (FE) studies showed that the implants made of PEEK material provided good experimental and clinical performances [15-20].

The finite element method, as an essential complement for the *in vitro* biomechanical studies, has been widely used for the study of lumbar spine [9-12,20-22]. However, the major deficiency of FE model of lumbar spine was the simplification of both the anatomic structures and material properties of ligaments.

Although the influences of fusion rate, and ASD on the range of motion, stiffness, flexibility of lumbar spine following the PLIF procedure with AIB [21] and PLIF with cages made of PEEK [20] or Ti materiel [9,10] have been investigated using FE method, respectively. To our knowledge, there are few studies evaluating the benefits or adverse effects among these three surgical scenarios using the model contained ligaments of three-dimensional (3D) solid elements. The aim of this study was to comparatively investigate the differences among three types of fusion construct, which may provide theoretical basis for the choice of a suitable surgical scenario for different patients.

## Materials and methods

### Establishment of the intact L1-S1 segment model

**Figure 1:**
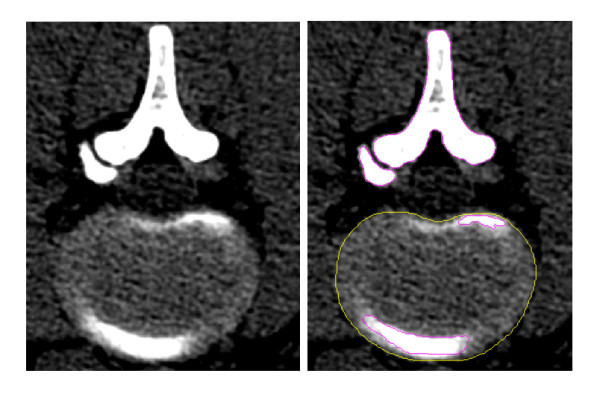
**Bony boundary and the disc outlines from DICOM image based on different gray value threshold.** (The region in the yellow outline was the disc and the region in the purple outline was bone structure.)

A 3D nonlinear FE model of L1-S1 segment that consisted of five lumbar vertebral bodies, one sacral vertebra, five intervertebral discs, and thirty-five spinal ligaments was developed using MIMICS and ABAQUS softwares. Geometrical details of all the parts in the model were obtained from computed tomography (CT) images with a slice distance of 2.5 mm (512 × 512 resolution, 8-bit, and a pixel size of 0.91 mm) of a 23-year-old male volunteer. CT data were imported into MIMICS software to establish six vertebral bodies, five annulus fibrosus (ANN) and five nucleus pulposus (NUC). Bony boundary and the disc outlines were depicted from each DICOM image filtered using a gray value threshold, which was shown in Figure 1.

**Figure 2:**
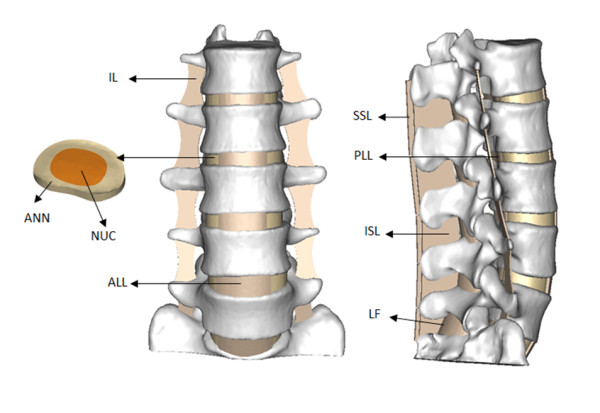
**3D** **model of spinal segment L1-S1.** ANN - annulus fibrosus, NUC - nucleus pulposus, ALL - anterior longitudinal ligament, PLL - posterior longitudinal ligament, IL - intertransverse ligament, LF - ligamenta flava, ISL - interspinal ligament, SSL - supraspinal ligament.

As the facet joints and ligaments were not seen clearly in the CT images, the facet joints and the surrounding ligaments, i.e., anterior longitudinal ligament (ALL), posterior longitudinal ligament (PLL), intertransverse ligament (IL), ligamenta flava (LF), interspinal ligament (ISL), supraspinal ligament (SSL) were modeled with four-nodal 3D tetrahedral (TET) elements according to their anatomical locations and morphologies. Each facet joint (FJ) was simulated by thirty spring elements. Figure 2 showed the 3D model of L1-S1 spinal segment.

Each part of the model was imported into ABAQUS software and the surface mesh was converted to volumetric mesh. The minimum edge length of the TET elements was 0.7 mm and the maximum edge length was 0.9 mm. The intact model totally contained 56 parts, 5596653 TET elements and 1067522 nodes.

### Establishment of the surgical models

#### A) simulation of the PLIF with cages

**Figure 3:**
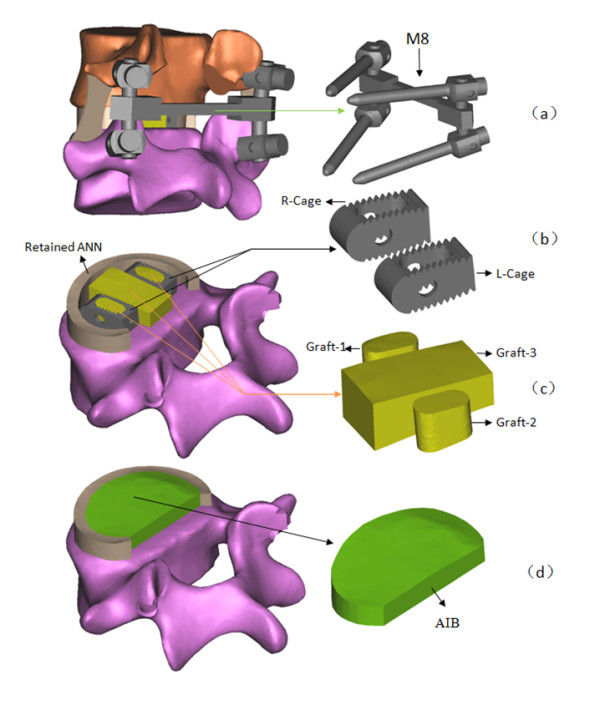
The implants of L4-5 surgical models, (a) M8 PSF, (b) cages, (c) bone grafts, (d) AIB.

The pedicle screw fixation (PSF), bone grafts and cages were modeled in SOLIDWORKS software. M8 PSF (Medtronuc SOFAMOR DANEK CD HORIZON TM M8) was used in this model. The length of the screws was 55 mm and the diameter was 5.5 mm. The M8 model was shown in Figure 3 (a). The material property of titanium (Ti) was assigned to the M8 PSF. The cage (Medtronuc SOFAMOR DANEK basis Cage) with 11 mm height was chosen since it provided a best fit across the L4-L5 disc space in the model. The established cage models were shown in Figure 3 (b).

The L4-L5 segment of the intact model was modified to simulate the PLIF procedure as shown in Figure 3. Two surgical models were established with Ti (E = 110 GPa) and PEEK (E = 3.5 GPa) material properties assigning to the cages of the PCT model and the PCP model, respectively.

#### B) simulation of the PLIF with autogenous iliac bone

All the modeling process of the PAIB model was the same as the modeling process of the PCT and PCP, except that the AIB was inserted between the vertebral bodies instead of the cages, as shown in Figure 3 (d).

The numbers of elements and nodes of the parts in the surgical models were listed in Table 1.

**Table 1:** The numbers of elements and nodes of the parts in the surgical models

	**M8**	**L-Cage**	**R-Cage**	**Graft1**	**Graft 2**	**Graft 3**	**AIB**	**Retained-ANN**
**Elements**	339012	172203	171826	72966	80307	18562	74665	22157
**Nodes**	67652	34284	34225	14013	15320	3787	14464	5009
**Type**	C3D4	C3D4	C3D4	C3D4	C3D4	C3D4	C3D4	C3D4

### Material properties

The material of the model can be divided into three types: bone, soft tissue, and surgical instrumentations. The material property of the bone structure was represented in MIMICS using some empirical expressions of the relationship among density, CT value, and Young’s modulus [23]. Since the gray values of thin cortical shell, inner trabecular core and endplates were different, the 200 kinds of different densities were calculated from the gray values based on the expressions. Then the 200 kinds of different elastic moduli were calculated from the above densities. This method would not only distinguish the material among the thin cortical shell, inner trabecular core and endplates, but also represent non-uniform material distribution.

The bone grafts were made from the mashed spinous process of L4, so the elastic modulus of bone grafts was low (E = 100 MPa) [10]. The iliac crest is mainly made up of cancellous bone, covered with a thin cortex. The material property of the iliac crest in this study referred to a previous study who defined the elastic modulus of the iliac crest as 1500 MPa [24].

As the visco-elastic property of the intervertebral disc and ligaments were not obvious in the quasi-static loading condition, the material properties of intervertebral discs and ligaments were modeled as hyper-elastic. The fluid-like behavior of the NUC and the ANN were both modeled with a hyper-elastic Mooney-Rivlin formulation [25,26]. The detailed parameters of the models and the material properties of surgical instrumentations were listed in Table 2.

**Table 2:** Summary of the material properties used in our finite element models

**Model**	**Material model**	**Material property**			**Literature**
**Bone**	Linear-elastic	*ρ* = 1.067 * HU + 131 (g/cm^3^)			23
		*E* = 0.09882 *ρ*^1.56^ (MPa)			
**Intervertebral disc**		Mooney-Rivlin formulation (MPa)			
		c_10_	c_01_	*D*_1_	
**NUC**	Hyper-elastic	0.12	0.09	1	25
**ANN**		0.56	0.14	1	26
**Ligaments**		Ogden-3 formulation (MPa)			27, 28
**ALL**	Hyper-elastic	(See Table 3)			
**SSL**					
**ISL**					
**PLL**	Hyper-elastic	(See Table 3)			
**LF**					
**IL**	Hyper-elastic	(See Table 3)			
**Facet Joint**	Linear-elastic	Spring elements: E=250 (MPa)			29
		Modulus of Elasticity	Poisson’s Ratio (μ)		
**Graft bone**	Linear-elastic	100 (MPa)	0.2		10
**AIB**	Linear-elastic	1500 (MPa)	0.3		24
**PEEK**	Linear-elastic	3500 (MPa)	0.3		20
**Titanium**	Linear-elastic	110 (GPa)	0.3		11

The stress–strain relationships of different ligaments were obtained from the experimental study [27]. The nonlinear behaviors of the stress–strain of ligaments were fitted by hyper-elastic Ogden-3 formulation in ABAQUS software [28]. The fitting results were listed in Table 3.

**Table 3:** The parameters of the fitting results for the different ligaments

	***μ***_**1**_	***α***_**1**_	***μ***_**2**_	***α***_**2**_	***μ***_**3**_	***α***_**3**_	***D***_**1**_	***D***_**2**_	***D***_**3**_
**ALL, SSL, ISL**	0.177	-3.080	0.627	-13.860	-0.357	-6.800	1	1	1
**PLL, LF**	0.159	-1.126	0.770	-18.540	-0.390	-9.600	1	1	1
**IL**	-6412.7	-7.3	4159.0	-9.0	2254.5	4.09	1	1	1

### Contact, boundary and loading conditions

The interaction property “TIE” in ABAQUS was used to define all the “surface to surface” contacts. The FJs were simulated as spring elements. The intact model contained 95 TIE interactions and 300 spring elements [29].

The nodes of the inferior surface of S1 were completely fixed in all directions.

To validate our intact model, pure unconstrained 10 Nm extension (e), 10 Nm flexion (f), 10 Nm lateral bending (l), and 10 Nm torsion (t) moments were applied to the superior surface of L1 vertebral body, respectively. Five load steps were applied to reach to the 10 Nm moments in each loading condition.

To validate the intradiscal pressure (IDP) of the intact model, the L4-L5 segment was modeled and calculated independently. The inferior endplate of the L5 vertebral body was rigidly fixed. Pure unconstrained moments of 10 Nm extension and flexion were applied to the superior endplate of the L4 vertebral body, respectively.

To compare the differences among the three surgical models under physiological loading condition, the surgical models were stressed with a 400 N of axial compression and 10 Nm moments to simulate extension, flexion, lateral bending and torsion. The intact model was also recalculated under the above loading conditions.

## Results

### Model validation

To validate our intact model, the FE results of range of motion (ROM) were compared with a previous *in vitro* experimental study under the same loading conditions [30,31]. As Table 4 shown, a good agreement was obtained between our numerical results and the reported data.

The numerical data in this study were within ± 0.8 standard deviation of the average of *in vitro* study. The results of the IDP were additionally compared with the data from previously performed experimental studies [32]. As shown in Table 5, the results of IDP in our FE model were within the range of the *in vitro* results. However, the majority of results in our FE model were larger than the average values of the *in vitro* results. This may due to the fact that there were no fiber-reinforced structures in ANN. It will be improved in our further study.

**Table 4:** **The comparison of the ROM between our FE model and the previous*in vitro*experimental study**

**Segment**	**Moment (Nm)**	**Extension and flexion**		**Left and right lateral bending**		**Left and right torsion**	
		***In vitro***	**FEA**	***In vitro***	**FEA**	***In vitro***	**FEA**
	2.5	7.5(1.35)	6.64	6.5 (1.4)	6.9	2.7(1.3)	3.62
L1-L2	5	8.3(1.3)	8.69	7.6 (1.35)	8.5	2.6(1.5)	3.04
	7.5	9.2(1.25)	8.93	8.9(1.2)	9.32	3.9(1.1)	3.47
	10	10.4(1.45)	10.2 9	10.2(1.5)	9.78	4(1.7)	4.02
	2.5	6.5(1.25)	7.62	8.8(1.2)	9.23	2.7(0.9)	3.24
L2-L3	5	10.1(1.2)	9.06	10.7(1.3)	11.2	3.15(1)	3.78
	7.5	11.1(1.2)	10.94	12(1.55)	13	3.9(0.85)	4.47
	10	11.5(1.3)	10.29	13(1.15)	13.43	5(0.95)	5.48
	2.5	8.3(1.75)	7.65	8.3(1.85)	8.43	2.5(1.1)	3.51
L3-L4	5	10.5(2.2)	8.93	10.5(2.15)	9.5	3.7(1.05)	4.27
	7.5	9.9(1.6)	10.24	11.4(1.5)	11.9	4.7(1.15)	4.79
	10	11.3(1.7)	1.78	12.2(1.7)	13.2	5.1(1.38)	5.76
	2.5	10(1.3)	11.25	7.5(1.45)	8.21	1.8(1.02)	2.57
L4-L5	5	12.5(2.25)	12.95	10.2(2.25)	10	2.4(0.85)	3.27
	7.5	14(2)	14.6	11.1(2.1)	10	2.8(1.2)	3.94
	10	14.8(2.1)	14.2	12.2(2.25)	13.23	3.7(1.5)	4.23
	2.5	13.5(3.3)	13.68	7.7(2.3)	8.2	1.5(0.55)	2.09
L5-S1	5	15.1(1.95)	16.17	9.3(2.15)	8.3	1.8(0.75)	2.35
	7.5	16.4(2)	17.66	10.2(2.2)	11	2(0.65)	2.48
	10	16.9(2.05)	17.29	11.3(2.35)	12.56	2.5(0.75)	2.7

(The ROM of each segment was determined by taking the sum of the two motions. The numbers in the brackets were the standard deviations.)

**Table 5:** **The comparison of the IDP between our FE model and the previous*in vitro*experimental study**

**Moment (Nm)**	**Extension**		**Flexion**	
	***In vitro***	**FEA**	***In vitro***	**FEA**
1	0.04 (0.03)	0.025	0.055 (0.035)	0.037
2.5	0.08 (0.07)	0.066	0.108 (0.05)	0.087
5	0.13 (0.11)	0.1335	0.18 (0.06)	0.198
7.5	0.16 (0.135)	0.198	0.27 (0.068)	0.312
10	0.17 (0.16)	0.262	0.35 (0.05)	0.384

(The numbers in the brackets were the standard deviations.)

**Figure 4:**
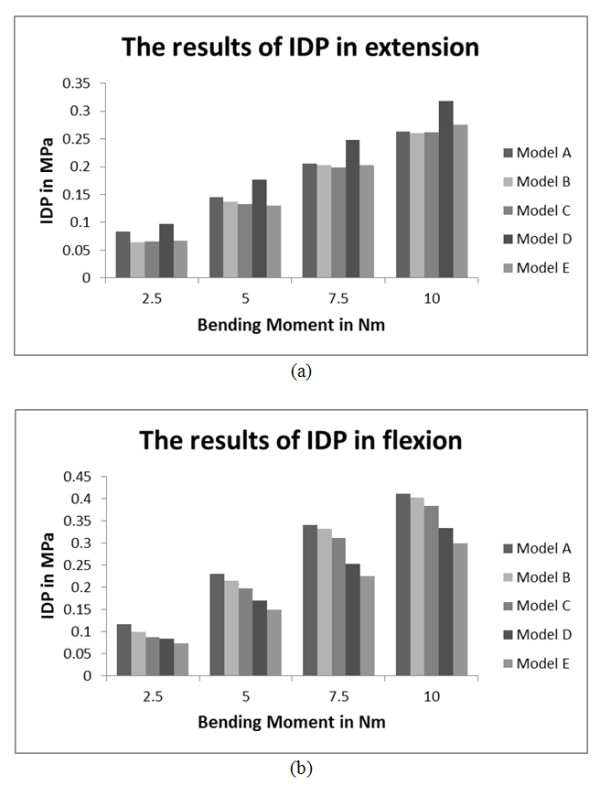
**The results of IDP of models A to E.** (**a**) the results of IDP in extension, (**b**) the results of IDP in flexion. (Models A to E were five remeshed L4-L5 models with different mesh densities. The detailed parameters of the mesh densities of these models were shown in Table 6.)

The model sensitivity analysis was also performed using the results of IDP. The L4-L5 segment was remeshed to establish models A to E. These five models were established with different mesh densities, as shown in Table 6. The results of IDP of the five L4-L5 models with different mesh densities were shown in Figure 4. The overall results of the model sensitivity analysis showed that the value of IDP declined with the mesh density decreasing. Compared with model A, the decreases of model B and model C were not obvious. However, the calculational time of model A was about 10 times that of model C. As the mesh density increased, the results of IDP in model D and model E were instable. The average increase of IDP in model D reached to 21.8%, compared with model A in extension. The average decrease of IDP in model E reached to 38.9% in flexion. So it was shown that the mesh density chosen in this study was reasonable.

**Table 6:** Element size, number of elements and calculational time of the model with different mesh density

	**Model A**	**Model B**	**Model C**	**Model D**	**Model E**
The edge length of the TET elements (mm)	0.4-0.5	0.5-0.7	**0.7-0.9**	0.9-1.1	1.1-1.3
Number of elements	3668172	1859589	**977162**	495715	362041
Calculational Time (h)	36.98	8.43	**3.84**	1.3	0.45

(The mesh density of model C was the one used in the intact model in this study.)

The biomechanical behaviors of the three surgical models were compared with those of the intact model, respectively.

### Stresses on the implants

The maximum stress on the M8 PSF was found to be 239.153 MPa in the PAIB model under lateral bending loading condition. The maximum stress on the M8 PSF was minimal in the PCT model, which was 93.3163 MPa under extension loading condition. The maximum stress on the M8 PSF in the PAIB model increased by 3.51% compared with the PCP model. The maximum stress on the M8 PSF in the PCP model increased by 3.76% compared with the PCT model.

There were obvious differences in the maximum stress and the average stresses on the cages of the PCT and PCP models. The maximum stress on cages of the PCT model was 3.6 times that of the PCP model, and the average stresses on cages of the PCT model was 3.36 times that of the PCP model, at the most.

### Stresses and strains on ligaments

The increase/decrease rates or percentage changes in this study were described by the following equation: increase/decrease rate or percentage change = (Data of surgical model - Data of intact model)/Data of intact model × 100%.

The maximum percentage changes of stresses on ligaments were shown in Table 7. It was shown that the changes of stresses on ALL and PLL in the PAIB model were slightly smaller than those of the other two surgical models using cages. However, for other ligaments, the changes in the PAIB model were greater than the other two models. This phenomenon became especially pronounced at ISL and SSL. The maximum percentage changes of stresses on the PAIB model reached to 67.31% and 72.31% for ISL and SSL, respectively.

**Table 7:** The percentage change of the maximum Von Mises stress on different ligaments among three surgical models

	**ALL (L5-S1)**	**PLL (L3-L4)**	**LF (L1-L2)**	**IL (L3-L4)**	**ISL (L5-S1)**	**SSL (L5-S1)**
PCT	44.75%	22.16%	1.78%	31.45%	8.26%	14.35%
PAIB	44.08%	16.63%	1.93%	39.04%	67.31%	72.31%
PCP	44.44%	22.70%	1.68%	31.40%	8.75%	14.71%

(Percentage change = (Data of surgical model - Data of intact model)/Data of intact model × 100%. The segments shown in the brackets were the positions that maximum change occurred.)

The percentage change of the average strains on different ligaments was shown in Table 8. The average strains on ALL and PLL of three models decreased. The maximum decrease reached to 19.02%, 17.09% and 18.83% for the PCT model, PAIB model and PCP model, respectively. However, the average strains on IL of three models increased obviously. The maximum increase reached to 23.72%, 30.88% and 23.68%, respectively. The average strains on LF increased obviously on PAIB only. The changes of the strains on ISL and SSL were different in three surgical models. The strains on ISL and SSL increased in PAIB model and decreased in PCT and PCP models.

**Table 8:** The percentage change of the average strains on different ligaments among three surgical models

	**ALL**	**PLL**	**LF**	**IL**	**ISL**	**SSL**
PCT	-19.02%	-7.46%	0.81%	23.72%	-4.01%	-5.15%
PAIB	-17.09%	-9.48%	5.93%	30.89%	2.27%	1.54%
PCP	-18.83%	-7.44%	0.86%	23.69%	-5.01%	-4.89%

(Percentage change = (Data of surgical model - Data of intact model)/Data of intact model × 100%.)

### Stresses of the adjacent discs

In general, compared with the intact model, the stresses on the disc at each segment of three surgical models showed an increasing trend in torsion and decreasing trends in other loading conditions, as shown in Table 9. The stresses on the discs just inferior to the fusion segment were significantly different in certain loading conditions. The maximum increase reached to 60.89%, 61.58% and 62.72% under torsion loading condition and the maximum decrease reached to 62.97%, 62.39% and 63.14% under lateral bending loading condition for the PCT model, PAIB model and PCP model, respectively.

**Table 9:** The percentage change of the maximum Von Mises stress on the intervertebral discs among three surgical models

		**PCT**	**PAIB**	**PCP**
Extension	L1-L2	-27.92%	-26.11%	-27.92%
	L2-L3	-34.31%	-31.34%	-34.20%
	L3-L4	-27.70%	-4.80%	-27.60%
	L5-S1	-35.70%	-5.65%	-35.70%
Flexion	L1-L2	-3.65%	-3.48%	-3.60%
	L2-L3	-20.79%	-19.68%	-20.59%
	L3-L4	-27.74%	-8.34%	-27.66%
	L5-S1	-41.67%	-11.27%	-41.41%
Lateral bending	L1-L2	-7.89%	-6.60%	-7.72%
	L2-L3	-9.15%	-7.76%	-9.03%
	L3-L4	-17.07%	-2.70%	-17.28%
	L5-S1	-62.97%	-62.39%	-63.14%
Torsion	L1-L2	13.50%	13.45%	13.49%
	L2-L3	6.64%	7.62%	6.67%
	L3-L4	8.18%	15.61%	8.36%
	L5-S1	60.89%	61.58%	62.72%

(Percentage change = (Data of surgical model - Data of intact model)/Data of intact model × 100%.)

### Stresses on endplates of surgical segment

The maximum stress on L4 inferior endplate was 20.83 and 15.76 times that of the intact model at the most in the PCT and PCP models, respectively. The maximum stress on L5 superior endplate was 3.67 and 2.09 times that of intact model, at the most. And the maximum stress on endplate of the PCP model was smaller than the PCT model in each loading condition. The stresses on the endplates in the PAIB model were smaller than the intact model with the maximum decrease rate of 86%.

### Stresses on bone grafts

The comparison about the average stresses on bone grafts of three surgical models was shown in Table 10. For the PCT model, the average stresses of each bone graft in each loading condition were smaller than the other two surgical models. The PCP and PAIB models got similar average stresses on bone grafts. The average stresses on bone grafts of the PAIB model was 1.29 times that of the PCP model, at the most.

**Table 10:** The average Von Mises stresses on bone grafts of three surgical models in each loading condition

	**Model**	**Extension**	**Flexion**	**Lateral bending**	**Torsion**
graft-1	PCT	0.122157	0.194958	0.016597	0.128982
	PCP	0.228447	0.409267	0.04922	0.288651
graft-2	PCT	0.147298	0.306561	0.144857	0.170196
	PCP	0.249845	0.571044	0.28286	0.328011
graft-3	PCT	0.20448	0.302199	0.105593	0.202865
	PCP	0.27222	0.507136	0.18066	0.337146
AIB	PAIB	0.292492	0.737651	0.296522	0.499107

## Discussion

Finite element analysis (FEA) is a sophisticated simulation method, and also an effective tool for elucidating biomechanics in the spine. In the biomechanical evaluations based on FEA, it is important to establish a model that can accurately reproduce the mechanical property of each part. Establishing such a model requires accurate data on anatomic structures and material properties [13]. However, since ligaments show complicated material properties and large deformation, it is difficult to establish an accurate model of ligaments in FEA. Many researchers used two-dimensional tension-only truss or cable elements to describe the function of ligaments [11,13,22,26,29]. In the present study, the surrounding ligaments were modeled with three-dimensional solid elements. The material properties of ligaments were simulated by hyper-elastic Ogden-3 formulation based on the experimental data. The validated results indicated that the model established in this study could effectively reproduce the mechanical behaviors of L1-S1 lumbar segment. In addition, another advantage of the model established in this study was that it could directly obtain the stresses and strains of the ligaments. The results may be useful to predict the chronic degeneration and disease of ligaments.

The intensive discussions among the three surgical scenarios were shown below.

### Stresses of M8 PSF

The largest maximum stress on the M8 PSF was found in the PAIB model, with the PCP model following and the PCT model being the least in each loading condition. The increase in the stresses on the M8 PSF may induce the increase in the risk of the breakage of PSF. The maximum stress on the M8 PSF was significant larger in lateral bending and extension than in flexion and torsion. Therefore, clinically the patients were recommended to avoid excessive lateral bending and extension movements in the process of treatment and recuperation.

#### Stresses of cages

As the Ti material was stiffer than the PEEK material, both the maximum stress and the average stresses on the cages of PCT model were larger than those on the PCP model, which indicated that the Ti material cages in the PCT models suffered more stresses concentration than the PEEK cages. The greater stresses on cages may increase the risk of fine motion and mote on cages. The fine motion and mote of cages would cause inflammation of the fused segment and have adverse effect on the fusion process. So the PCT model was obviously inferior to the PCP model in this respect.

### Stresses and strains on ligaments

Compared with the intact model, the stresses on ligaments of the three surgical models increased significantly and the maximum increase of the stresses located at the segments that proximally adjacent to fusion segment (See Table 7). The greater stresses on ligaments were found in the PAIB model than the other two models. The maximum stress on PAIB model was about 8 times that of the PCT model and 5 times that of the PCP model, at the most.

The PAIB model also produced larger strains on the majority of ligaments. The ligaments were pre-stressed due to the increase of strains on ligaments, which reduced the ability of ligaments to resist stretching. The ligaments would be injured or fragmented more easily, when there is external load applied on the spine. The increase of stresses and strains on the ligaments also changed the normal physiological and mechanical environments of ligaments. These changes were likely relevant to the invocation of early pain and prone to cause chronic soft tissue injury and degeneration. In this respect, the PCP and PCT models were better than PAIB model. To our knowledge, there were few reports describing the stresses on ligaments in the PLIF procedure.

### Stresses of the adjacent intervertebral discs

Both the postoperative following-up and biomechanical studies showed that the PLIF accelerated degeneration of adjacent segment and segmental instability [11]. The FE results showed that great changes were found in the stresses on the discs proximally adjacent to the fusion segment. These great changes in discs could be used to interpret the clinical findings of early degeneration of adjacent disc [12]. The increase of the maximum Von Mises stresses on adjacent discs during torsion was probably due to the following reasons: the M8 PSF restrained more ROM than other loading conditions. Thus, the ROM of the adjacent segment increased a lot. And the Von Mises stresses on adjacent discs also increased. The decreases of the stresses on the discs in the PAIB model were smaller than the other two surgical models, especially at fusion adjacent segments under extension, flexion and torsion. This result showed that the surgical method using AIB could decrease the risk of degeneration of fusion adjacent discs.

### Maximum stress on the endplate of surgical segment

Of all the structures, the most significant changes in the maximum stress occurred on the L4 inferior endplate and L5 superior endplate. There were two main reasons that caused the tremendous increase in the stress on the endplates. Firstly, although the jagged edges of the cages avoided the relevant moments between endplates and cages, this design resulted in stress concentration. Secondly, the materials of PEEK and Ti were much stiffer than the bone grafts. So the phenomenon of stress shielding on cages was serious. The majority of the load was transferred onto the cages instead of the bone grafts. So the PLIF with cages caused tremendous increase in the stress on the endplates. Excessive stresses on the endplate may cause osteolysis of the endplate and subsidence of the fused segment. Compared with the surgical model using cages, the surgical model using AIB could reduce the stresses concentration on the endplates obviously, thus protect the endplate of the surgical segment.

From the results of stresses on the adjacent discs and the endplates, it was shown that the PAIB model was better than the other two models. Therefore, this surgical method was recommended for the elderly patients who had already suffered from the ASD and osteoporosis. This was because that the two major risks faced in the PLIF procedure were the further degenerative diseases of surgery adjacent segments and the subsidence or damage of endplate, which would eventually result in the failure of fusion surgery. Compared to the two models using cages, the PAIB model could effectively abate such phenomenon.

### Average stresses on bone grafts

**Figure 5:**
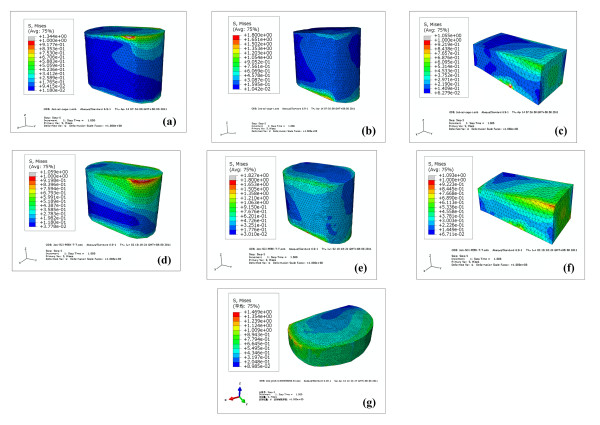
**Contour plots of Von Mises stresses on bone grafts for each surgical model in torsion**. (**a**) the graft-1 in the PCT model, (**b**) the graft-2 in the PCT model, (**c**) the graft-3 in the PCT model, (**d**) the graft-1 in the PCP model, (**e**) the graft-2 in the PCP model, (**f**) the graft-3 in the PCP model, (**g**) the AIB in the PAIB model.

The ultimate purpose of the PLIF was to complete bone graft fusion, restoring the height of intervertebral space and finally achieving long-term stability of the lumbar spine. Therefore, the fusion rate of the bone grafts was the key point of the surgery, and it was also the issue that our study focused on. According to Wolff’s Law, bone can change its structure according to its mechanical environment. So the stresses on grafts may be used to predict the long-term fusion rate [20]. As our FE results (see Table 10) shown, the PCP and PAIB models got similar average stresses on bone grafts, and were both larger than the PCT model. This was mainly due to the low stiffness of AIB and PEEK material, which reduced the stresses shielding on bone grafts. Therefore, the average stresses on the bone grafts of the PCP and PAIB models were significantly larger than those on the PCT model. The contour plots of Von Mises stresses on the bone grafts were shown in Figure 5. It can be seen that the stresses of the PCT model mainly concentrated on the inferior or superior surface of the grafts, whereas not so much stresses traversed the central part of the grafts (see Figures 5a5b and 5c). On the other hand, the stresses distribution of the grafts in the PCP and PAIB models was more extensive (see Figures 5d to 5g), which may better facilitate the fusion from grafts to endplate, and improve the efficiency of bone graft fusion.

To conclude, the comparative results showed that the greatest stresses on cages and endplates were found in the PCT model, but the stresses on the bone grafts were found lowest in this model, so it may be inferior to the other two surgical models. The PCP model and PAIB model showed a similar considerable stresses on the bone grafts. However, the PCP model showed a decrease in the percentage change of ligaments and less stresses on PLIF. The PAIB model showed a decrease in the percentage change of adjacent discs and lower stresses on endplates.

There are certain limitations in this FE study. Firstly, The ANN was modeled with an isotropic hyper-elastic material model without the fiber-reinforced structure. Secondly, the facet joints and capsular ligaments were simplified to 30 spring elements. Under the actual condition, the structures are more complex. Thirdly, muscle contractions were not simulated in the current study. The muscle contractions may bring complicated external forces that have significant influences on the biomechanical perspective [9]. The above factors will be considered in our further study. Although there were certain simplifications in our FE model, the FE model was well validated by the previous *in vitro* study. Therefore, the model established in this study is reasonable and can be used as an efficient tool to evaluate the effects of three surgical scenarios on the lumbar spine.

## Conclusions

The PCT model may be inferior to the other two surgical models. Therefore it was not recommended to use cages made of Ti material in an instrumented PLIF. Both the PCP model and PAIB model had their own relative merits, so the doctor could choose the most suitable surgical method based on the finding in this research for the different clinical circumstances.

The modeling method using the ligaments with 3D solid elements can be extended to other body parts such as knee joint, ankle joint and shoulder joint, in which the ligaments play an important role. The model can also be used as the basis for our further study, in which surgical models having cages with different shapes and grafts will be developed. Besides, bone remodeling theory will be introduced to predict the long-term bone graft fusion, which could provide theoretical basis for clinical postoperative rehabilitation.

## Competing interests

The authors declare that they have no competing interests.

## Authors’ contributions

XZT established the finite element model and drafted the manuscript. WLY participated in the modeling of the basic model and helped to finish the analysis of the results. GH conceived of the study, and participated in its design and helped to draft the manuscript. ZD provided the CT images and surgical implants. All authors read and approved the final manuscript.
